# Alternative starting regimen with aripiprazole long-acting treatments, a case report

**DOI:** 10.1192/j.eurpsy.2022.1866

**Published:** 2022-09-01

**Authors:** M. Huete Naval, B. Serván, M.E. Expósito Durán, P. Albarracin, E. Herrero Pellón, R. Galerón

**Affiliations:** 1 Hospital Clínico San Carlos, Institute Of Psychiatry And Mental Health, Madrid, Spain; 2 Hospital Universitario Clínico San Carlos, Psychiatry And Mental Health, Madrid, Spain; 3 Hospital Clínico San Carlos, Instituto De Psiquiatría Y Salud Mental, Madrid, Spain

**Keywords:** Aripiprazole, long-acting treatments

## Abstract

**Introduction:**

Aripiprazole long-acting treatments can significantly control symptom, improve adherence and reduce the risk of relapse compared to oral drugs. An alternative start-up guideline has recently been approved in several countries that simplifies its administration.

**Objectives:**

To present a case report of a patient with schizophrenia treated with alternative starting regimen of aripiprazole long-acting treatment.

**Methods:**

Presentation of a clinical case supported by a non-systematic review of literature.

**Results:**

We present the case of a 22-year-old patient diagnosed with schizophrenia, whose symptoms started after the birth of her son, 2 years ago. She has presented a poor clinical evolution, requiring several admissions to our inpatient service after discontinuation of her medication. The patient has taken different antipsychotics, including olanzapine and paliperidone long-acting treatment, which were suspended due to side effects (weight gain and increased prolactin levels). A switch to oral aripiprazole 20mg was made, which showed good response and tolerance. Given the persistence of irregular intake, it was decided to switch to aripiprazole long-acting treatment, applying an alternative initial regime consisting of two doses of aripiprazole long-acting treatments 400mg and one oral aripiprazole 20mg. The patient has since had no delusions or hallucinations and is living independently at home.

**Conclusions:**

The administration of a simplified initial regime with aripiprazole long-acting treatments could improve therapeutic adherence while maintaining the same effectiveness and similar side effects.

**Disclosure:**

No significant relationships.

